# Self-management of overactive bladder at home using transcutaneous tibial nerve stimulation: a qualitative study of women’s experiences

**DOI:** 10.1186/s12905-021-01522-y

**Published:** 2021-10-27

**Authors:** Ciara M. E. Daly, Lynette Loi, Jo Booth, Dalia Saidan, Karen Guerrero, Veenu Tyagi

**Affiliations:** 1 Department of Urogynaecology, Queen Elizabeth University Hospital, Glasgow, Scotland, UK; 2 School of Health and Life Sciences, Glasgow Caledonian University, Glasgow, UK

**Keywords:** Urinary bladder, Overactive, Transcutaneous electric nerve stimulation, Tibial nerve stimulation, Qualitative research

## Abstract

**Background:**

Transcutaneous tibial nerve stimulation (TTNS) has been used to treat overactive bladder (OAB), however patient experiences and views of this treatment are lacking. The aim of this study was to explore women’s experiences of OAB and TTNS treatment and the perceived factors influencing participation and adherence.

**Methods:**

Semi-structured, individual interviews conducted as part of a mixed-methods, randomised, feasibility trial of self-managed versus HCP-led TTNS. Interviews were audio recorded and transcribed verbatim. Reflexive thematic analysis was undertaken using Booth et al. (Neurourol Urodynam. 2017;37:528–41) approach.

**Results:**

16 women were interviewed, 8 self-managing TTNS at home and 8 receiving TTNS in twice-weekly hospital clinic appointments. Women self-managing OAB considered TTNS easy to administer, flexible and favourably ‘convenient’, especially when the participant was bound by work and other life commitments. In contrast to OAB symptoms ‘dominating life’, self-managing bladder treatment was empowering and fitted around home life demands. Flexibility and control engendered by self-management, facilitated women’s willingness to participate in TTNS. Women attending a hospital clinic for TTNS enjoyed the social aspects but found the routine appointments constrained their lives. Motivation to continue TTNS in the longer term was dependent on perception of benefit.

**Conclusions:**

This study provides novel insights into women’s experiences of self-managing their OAB using TTNS compared to HCP-led management in the clinical setting. It highlights positive experiences self-managing TTNS at home and a willingness to continue in the longer term, facilitated by ease of use and convenience.

*Trial Registration* 1/11/2018: ClinicalTrials.gov Identifier: NCT03727711.

## Background

Overactive bladder (OAB) syndrome is defined as urinary urgency, usually accompanied by frequency and/or nocturia, with or without incontinence [1]. First line conservative treatments include: lifestyle advice, bladder training, pelvic floor exercises in mixed urinary incontinence and vaginal oestrogen when vaginal atrophy is present in postmenopausal women. A meta-analysis has shown 40% of people with OAB do not achieve acceptable therapeutic benefit with anticholinergic medication and side effects influence willingness and motivation to continue with treatment [2]. Transcutaneous tibial nerve stimulation (TTNS) is a non-invasive, neuromodulation treatment for OAB. It is thought to inhibit bladder afferent activity through interneurons activated by somatic sensory pathways originating in the lower limb via the tibial nerve [3]. A systematic review of 10 RCTs and 3 prospective cohort studies involving 629 participants, showed 48–93% significant symptom improvement following a programme of TTNS [4]. TTNS involves application of reusable skin surface electrodes and electrical stimulation of the tibial nerve as a programme of treatment sessions (usually 12) [4]. A full treatment programme requires time and travel commitments for delivery in hospital clinics. Self-management at home offers the potential for TTNS to be a cost-effective alternative to the minimally invasive Percutaneous Tibial Nerve Stimulation (PTNS) which delivers stimulation via single-use needles inserted by a healthcare practitioner [5]. Side effects of PTNS include bleeding and/or pain at the site of needle insertion which the transcutaneous route avoids [6]. A recent prospective RCT (n = 60) compared Bladder Training (BT) alone to PTNS plus BT and TTNS plus BT. Severity of incontinence, frequency of voiding, incontinence episodes, nocturia, number of pads used, symptom severity and quality of life were significantly improved in the TNS groups versus BT alone (*p* = 0.0167) [7]. TTNS had shorter preparation time, less discomfort level and higher patient satisfaction than PTNS.

There are also significant equipment costs associated with PTNS. In the first year of use it has been estimated that PTNS costs $3199, reducing to $2012 for 15.6 PTNS treatments per year in subsequent years; this is comparable with total costs of antimuscarinic therapy [8]. Home TTNS is much more economical due to the minimal equipment costs (commercially available Transcutaneous Electric Nerve Stimulator (TENS) and 4 surface electrodes per treatment programme; estimated at £77 in 2020) [9]. TTNS could be a useful adjunct to first-line conservative treatments.

Understanding patient experiences of TTNS is lacking and there are no published studies exploring treatment contexts or model of delivery. The study reported here is part of a mixed-methods, randomised, feasibility trial comparing TTNS self-management at home with clinic based, health care professional led (HCP-led) TTNS for OAB management in women. The aim of the study was to explore women’s experiences of OAB and the TTNS treatment and perceived factors influencing participation and adherence. Feasibility trial results and cost-effectiveness analysis will be reported separately.

## Methods

We used a critical realist orientation [10] to guide the qualitative investigation in the mixed methods design, focussing on understanding the meaning of living with OAB while recognising the women’s real-world experiences of these conservative treatments to elicit underpinning explanatory mechanisms. Participants were women seeking treatment for OAB symptoms. All were on the first-line conservative/OAB medication management pathway and were recruited from outpatient urogynaecology clinics as part of the feasibility trial (TPoTS trial: an RCT comparing Home versus Hospital TTNS Treatment for OAB ClinicalTrials.gov Identifier: NCT03727711). Urodynamics were not part of the study requirements. Trial eligibility criteria are shown in Table 1.

**Table 1:** Eligibility criteria

*Inclusions*
1. Women aged ≥ 18 years
2. Clinical diagnosis of OAB
3. Post-void residual < 100 ml
4. Able to complete questionnaires and interview
5. Willing to consent to participate
iExclusions
1. Cardiac pacemaker in situ
2. Leg ulcer/skin condition affecting lower legs
3. Diagnosed peripheral vascular disease
4. Absent sensation at the electrode site
5. Active UTI
6. Pregnancy
7. Previous PTNS/SNS
8. Previous intravesical botox treatment
9. Stress Urinary Incontinence as the predominant symptom
10. New treatment for OAB/incontinence commenced in the 4 weeks prior to TTNS

*OAB* overactive bladder, *UTI* urinary tract infection, *PTNS* percutaneous tibial nerve stimulation, *SNS* sacral nerve stimulation, *TTNS* transcutaneous tibial nerve stimulation

Participants were randomised to TTNS self-management at home or hospital HCP-led TTNS. Women in the hospital clinic group attended an outpatient clinic to have the TTNS treatment administered by an HCP. Women randomised to self-manage at home, were taught to self-administer the TTNS treatment. This involved a single session involving individual demonstration of TTNS application and treatment technique, followed by supervised guidance by research nurses to ensure the woman’s competence to correctly apply and adjust the TTNS. Women were provided with all equipment, written instructions and details on who to contact if they encountered difficulties during the intervention period.

**Fig. 1:**
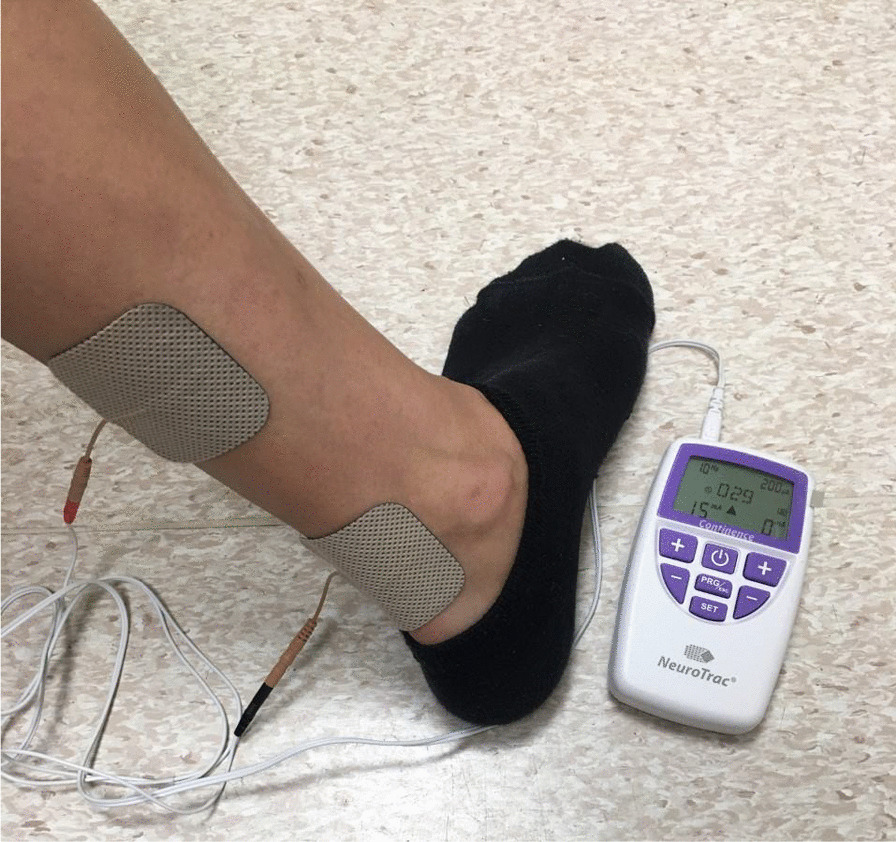
The NeuroTrac Continence machine stimulation parameters and surface electrode positioning

For both groups the TTNS programme involved delivery of 30-min stimulation sessions twice weekly for 6-weeks, using the NeuroTrac Continence machine—model NT4 (CE marked). Two reusable surface electrodes (50 × 50 mm gel pads) were applied to the right ankle: the cathode placed behind the medial malleolus and the anode approximately 10 cm cephalad to this (Fig. 1).

Verification of the hallux movement in response to the electrical stimulus was performed to confirm stimulation of the tibial nerve. The stimulation parameters were standardised (pulse frequency 10 Hz, pulse width 200 µs [11]) with the intensity adjusted according to the woman’s individual comfort (to a maximum 50 mA). Women were allowed to move about freely at their preference during TTNS delivery. Participants self-managing at home were contacted by telephone at 3-weeks to check concordance with the treatment programme and discuss any issues.

All women were approached for individual interview during recruitment into the main feasibility trial and informed consent obtained. Following completion of their 6-week TTNS treatment programme the women were contacted by telephone to confirm consent and to answer any questions. A purposive sample of eight participants from each study arm was recruited based on serial order of recruitment to the trial. Face-to-face or telephone interview was scheduled, according to preference. A flowchart of recruitment into the qualitative study is available in Table 2 below.

**Table 2:**
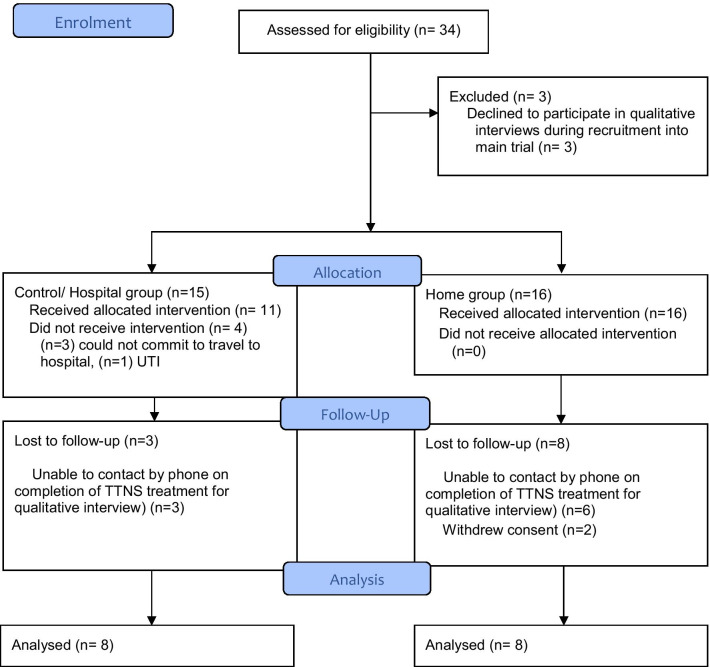
Participant recruitment flowchart

*TTNS* transcutaneous tibial nerve stimulation

All interviews were completed during May and July 2019 by a researcher who was not involved in the TTNS treatment delivery (CD) at the research centre. (CD) undertook two pilot interviews with the supervising researcher (JB) to learn the techniques and gain feedback on interview style. Only the interviewer (CD) and participant were present during interviews. Interviews were semi-structured and addressed the topics detailed in Table 3. Each was audio-recorded, transcribed verbatim and imported to NVivo 12.5.0 for data processing and analysis. Patient identifiers were anonymised in the transcripts. All methods were performed in accordance with the relevant guidelines and regulations in the methods section. No repeat interviews were performed. The only other involvement/ contact was for any incomplete follow-up bladder questionnaires as part of the quantitative study. South Central Berkshire B Research Ethics Committee (18/SC/0053) approved this study.

**Table 3:** Topics explored during qualitative interviews

1	Woman’s perceptions of urinary symptoms and how they affected her prior to TTNS treatment and since completing treatment
2	Views about self-managing bladder symptoms in general e.g. fluids, anticipatory toileting, toilet mapping, restricting activities etc
3	Expectations of TTNS e.g. how it would feel, impact on lifestyle/ daily routine, anticipated effects
4	Experiences of the TTNS programme (self-managed at home and HCP-led in hospital settings)
5	Factors affecting TTNS use and adherence (enablers and barriers)
6	Any changes in bladder (self) management approaches following TTNS treatment
7	Views about self-managing with TTNS and considerations about bladder health in the future

*TTNS* transcutaneous tibial nerve stimulation, *HCP* healthcare professional

Reflexive thematic analysis was completed using Braun and Clarke’s [12] five stage approach. Data familiarisation began with the lead researcher (CD) undertaking all interviews, reading and re-reading transcriptions to create initial reflexive thoughts and ideas about the meaning of the data. Initial codes were generated and were further reviewed and refined with the senior researcher (JB). Codes were organised into initial themes by, separating, combining, discarding and collating codes into concepts with similar meaning in the iterative refinement process. The thematic analysis focussed on the identification of patterns across data, interpretation and theme generation from the interviews undertaken by purposive sampling. Two researchers agreed the interpretation and the themes and concluded that the sampling yielded a rich description of the patient experience. These initial themes were reviewed, developed and further refined during the ongoing analytic and reflexive processes [12].

## Results

16 qualitative interviews were completed with 8 women who were self-managing their TTNS and 8 women who were attending weekly hospital clinics to have TTNS applied by an HCP. The median interview duration was 18 min (range 11–33 min). Participant mean age was 61 years (range 38–78 years).

The analysis generated four themes:

1. Losing bladder control is a part of everyday life
2. Self-management versus HCP-led treatment: Just because you’ve a bladder problem, doesn’t mean your life stops
3. TTNS is both a physical and a mental treatment
4. Preferences for OAB treatment: Am I bad enough?

### Losing bladder control is a part of everyday life

Throughout the analysis the women repeatedly referred to OAB impacting detrimentally on their lives. They talked about persistently losing control over their bladder across life situations and that living with OAB meant trying to achieve bladder control when symptoms are unpredictable and come with little warning. The loss of control they experienced included physical bladder symptoms of urgency and leakage but they also highlighted the unpredictability of when it might happen and the extent of the leakage as having a major impact across social interactions, relationships and daily activities.

“The, not knowing… if it will be just a dribble, a spot.. or a.. flood.”

Participant 13 (Self-managing)

“I am a member of admin and I'm supposed to take minutes as well and that was affectin' me because there were times that I have to say, 'I'm really sorry I need to nip out for a minute'. I mean I would have a table of people waiting for me to come back in again, so that could become quite embarrassing really.”

Participant 10 (HCP-led)

“Erm… it’s just you know… the bladder leakage. It’s just a part of life. It has been for a long, long time.”

Participant 7 (Self-managing)

Loss of bladder control impacted on women’s self-esteem and their confidence in their ability to control their bladder. Home was perceived as a safe space and when unpredictable OAB symptoms occurred, access to a toilet was close-to-hand. However, when venturing outside the home setting, successful bladder management was more difficult. Women felt nervous as it involved an element of risk-taking. The impact of their unpredictable and uncontrollable OAB symptoms on self-esteem influenced motivation to regain bladder control and achieve ‘normality’, both in their activities outside home and in how they viewed themselves.

“So if I'm a-, if I'm at home and I haven't got to toilet in time or I'm just getting to toilet in time and I have a leak, it's fine because I can just freshen up and change my underwear. But if you're out about and that happens… you could just become really self-conscious..’em, spray perfume (laughs)”

Participant 16 (HCP-led)

“Well, I’m willing to try anything to stop this. As I said, I’m 41. It’s ridiculous to be.. I feel like an old granny.. and eh, I’m not enjoying my life to the fullest capacity. I shouldn’t be needing to be wearing these big giant pads, erm.. say if I’m going out at night, if I drink alcohol, I would have to wear a giant pad, because then, you don’t know how long you gonna be waiting for a taxi or if you(‘re) gonna make it home”

Participant 6 (Self-managing)

### Self-managing versus HCP-led treatment: Just because you’ve a bladder problem, doesn’t mean your life stops

Home treatment was viewed favourably as ‘convenient’, especially if the participant was bound by work commitments.

“So coming into the hospital to get that (TTNS) done, would be an inconvenience to me unless, it could be done, you know, at half past four.. after work, whereas in the house, it’s MUCH more convenient.. MUCH more convenient. You know, really I just think it’s.. yeah.. it definitely works.. but the convenience of doing it at home I just think it would benefit a lot of people… including like myself. Just because you’ve (a) bladder problem doesn’t mean your life stops.”

Participant 7 (Self-managing)

The flexibility of self-managing facilitated adherence as women could choose the times and days of treatment to fit around their activities and there was no requirement for a fixed timetable other than twice weekly TTNS sessions. Thus self-adjustments could be made to accommodate the woman’s individual lifestyle and needs.

I thought it was useful to know I didn't have to do it on a specific day of the week, that you could do two days in a row or two alternate days.. as long as two fell within the week. So it was handy to do that because sometimes, life caught over and… I wasn't, you weren't able to do it when you had initially planned.

Participant 23 (Self-managing)

The women continued household routines whilst using TTNS, thus the impact on their daily routine was minimal. This was a very important consideration for women because unlike OAB symptoms dominating ‘life’, bladder treatment could fit in around ‘life’. This was perceived as empowering and facilitated willingness and motivation to participate in TTNS treatment. Walking about during TTNS distracted from awareness of stimulation and allowed women to increase the intensity to their desired setting.

Family support for TTNS was viewed as a positive facilitator. Some women who were self-managing at home did state the need be reminded to use the machine. Having a dedicated time for treatment was thought of as a way to facilitate treatment concordance.

And I used to just sit and watch TV or walk about and do some housework. I found if I walked about, obviously the machine, the way that it works… I didn't feel it much so I could turn (it) up higher…”

Participant 3 (Self-managing)

“Well, mostly in the afternoon I would say (I used it), and maybe in the evening if I’d been out in the afternoon, I used it. My husband kept me right as well and he kept on saying “use your machine!” (laughs)”

Participant 2 (Self-managing)

In the HCP-led hospital group, positive and negative experiences were seen. Women felt they had set appointments and enjoyed a social aspect. Where transport links were good, they had no problem attending but when this was not the case, hospital treatment was an inconvenience to their daily schedule.

“Aye. It was fine (hospital setting). The staff are amazing, they are very nice. I think I kept them more entertained than anybody else with the stories I was tellin’ about… (laughs). I was quite fortunate that the first bus runs from the bottom of my road and it drops you just up at the stop…”

Participant 1 (HCP-led)

“Sometimes I drove a- and sometimes I just took the bus because it was murder trying to get parked.”

Participant 12 (HCP-led)

### TTNS is both a physical and a mental treatment

There was a sense of regaining control when benefit was seen with TTNS in both the self-management and the HCP clinic groups. Improvement in bladder condition was subtly noticed by participants and close family members. Even if improvement did not mean complete resolution of symptoms, the effect was noteworthy and appreciated as a sense of regaining normality by the women.

“See by, I would say by the fifth week, erm… even my husband says, ‘Is there something wrong with ya?’ He says, ‘I’m not waking up when you..’ I mean it's like Blackpool illuminations.. I've got all the lights on you know, and of course it wakes him, and I says ‘no, I’m not waking up during the night to go to toilet’ and my pad was quite dry.”

Participant 4 (HCP-led)

“Well I think just because I can go when I want to go instead of out runnin' when I need to go, rather than when, you know.. just making (it on) time.. has made an awful difference.”

Participant 15 (HCP-led)

However, the improved physical symptoms were complemented by increased mental confidence and psychological benefits for many. Most accepted this readily and felt comfortable with the effects gained, whereas others considered whether the positive effects experienced would continue if they were ‘tested’ e.g. if they had to hold off for the longer before reaching a toilet, although the perceived benefit overall was taken as positive.

“I didn't know that I would feel this tingling which is immensely soothing.. for somebody with a bladder problem, it just.. it.. relaxes my bladder, can I tell you… that's what it does, it relaxes your bladder and it makes your mind feel better”

Participant 9 (Self-managing)

“…I was okay, I mean, I got to the toilet-, and I-, got to toilet and probably on time… I mean if I had been much longer, maybe I would've had a leak-, but ‘em, I got to toilet in time so I'm.. I'm feeling better.. not so upset (laughs).. not so well, I'm really.. maybe not so worried..”

Participant 16 (HCP-led)

Some wondered if the benefits they felt were real or ‘in their mind’ but rationalised this by describing better physical control in toileting and reduced anxiety overall—again, this did not deter from the satisfaction gained overall.

“Yes I did think I benefitted from it quite significantly, ‘em I don't know whether a lot of it is in your mind but.. but I had that wee added security that, that I could manage to get to the toilet in time without having to panic about it.”

Participant 3 (Self-managing)

### Preferences for OAB treatment: am I bad enough?

Regardless of whether it was self-managed or HCP-led, TTNS was strongly favoured as a management approach for OAB symptoms when positive treatment response was reported. There were opinions expressed that TTNS would need to be continued in the long-term to maintain the benefits achieved but this was seen as acceptable by the women in both groups, if symptoms were controlled.

“Well they're portable, they're lightweight, they don't use up a lot of energy.... there's not really anything that would stop me from using it again”

Participant 13 (Self-managing)

“I do think that things have improved. I do think that this has helped. Absolutely. I'm keen to see what the next weeks bring in because I think I'm probably converted to-, to usin' this, thereafter.”

Participant 16 (HCP-led)

Even with sub-optimal treatment response, there was a perception that effects may accumulate over time and continuing for longer may prove beneficial. However, financial considerations were influential for some.

“I mean, don’t get me wrong, I would use it, because it didn’t make me any worse, and it might have made me slightly better. So if I was given the machine, I would use it. But I don’t think the improvement is big enough to actually buy one, it didn’t show a massive improvement. As I was saying, I don’t know if I kept going, if it might have improved me more.”

Participant 5 (HCP-led)

“I would’ve kept on it (TTNS), but it felt.. (SIGHS).. the cost of it was too expensive”

Participant 4 (HCP-led)

Women self-managing using TTNS at home were more strongly aligned to continuing treatment and expressed high satisfaction for this form of treatment for OAB.

I would think about it by itself (TTNS), ‘cause it did work by itself. But I wouldn’t think about it unless I could do it at home…… because coming to the hospital is an inconvenience, it will get in the way, in my life.”

Participant 9 (Self-managing)

Participants who attended the HCP-led clinic were open to future treatment in either setting but felt confident they could self-manage at home having watched the application of the electrodes by HCPs during their treatments.

“Basically, it was done through the hospital… the two pads (went) on my ankle and then basically you know, ‘em you know, the first time.. the (research nurses) were tryin' to get to the level I was comfortable with, you know ‘em, ‘til I felt the pulse..”

Participant 10 (HCP-Led)

“so I now use the machine in the house, I've bought it and that's keeping my symptoms at bay”

Participant 10 (HCP-Led)

This confirmed the experiences of those women self-managing TTNS at home who considered the treatment easy to administer and were keen to have future treatments at home. They found home treatment easy to administer and safe to use without limitations.

“As I said, it was really easy to do, I’ve got to say, it was literally just sticking the pads on and sticking it on, and just turning it up.”

Participant 9 (Self-managing)

“I think it's quite a simple machine to use.”

Participant 13 (Self-managing)

“I used to just put it on and sit and maybe watch.. eh.. the television. When it was on obviously it was timed and it will go off on its own accord, so there was no problem with it at all.”

Participant 6 (Self-managing)

Uncertainty about impact of complications from more invasive OAB treatments and how these could be managed was evident and shaped by previous experiences. This uncertainty centred around how OAB treatment complications could impact on everyday life and reinforced the women’s’ desire to continue with a more conservative approach.

“No no, I don't like the sound of that (Botox) to be honest. I'm just a bit fearful about that kinda.. well I feel as though I'd be the one in ten that would need to.. use a catheter and then.. how does that impact when you're working? Would I not be working? Would I need to let (others) know about my condition? uuggh, I'm not so keen on that one.”

Participant 16 (HCP-led)

This was in contrast to TTNS where women were willing to ‘try it’ for longer, even where response was suboptimal as it was viewed as a safe, non-invasive, pain-free treatment they could self-manage.

Preferences to continue TTNS as part of a self-management strategy were shaped by perceived treatment benefit, duration of effect, availability of home-setting and affordability of the treatment.

## Discussion

This study adds novel insights on women’s views about using TTNS to treat OAB and their reflections on self-managing in the home setting compared to HCP-led clinic-based treatment. The desire to try TTNS is encouraging and self-management by women is feasible and acceptable and the preferred option, where benefit was seen. Working women particularly found self-management at home convenient, flexible and empowering, allowing life to continue whilst trying to regain bladder control.

In a qualitative review [13] evaluating the factors contributing to therapeutic non-compliance, factors were grouped into the following categories: patient-centred, socioeconomic, disease-related, healthcare system factors and therapy-related.

Patient-centred factors affecting adherence were seen in this study and were mainly psychosocial. Beliefs regarding sub-optimal efficacy were divergent; some discontinued whilst others were keen to give the conservative treatment the ‘best shot’ and use it for longer. Regarding motivation, most had no problems but some used family support to aid adherence. The benefit of social interaction was common to both home and HCP-led treatment. Our findings correlate with those from other studies [14–17]; patients who have emotional support and help from family members, friends or healthcare providers are more likely to be compliant to the treatment. A combination of reducing negative attitudes and increasing motivation, as well as remembering to implement the treatment are the benefits. Involving family members in discussions about the treatment course could be a good way of encouraging adherence further.

The economic factors of affordability and inability to take time off work came to light in this study. Cost is a crucial issue in patient’s compliance especially where long-term treatment is required [18]. We saw discontinuation when participants had to purchase their own machine at the end of the study despite having ‘disease improvement’. Participants were recruited from government-funded medical and health care services—the National Health Service (NHS). Should TTNS become a standard treatment in the conservative OAB management pathways, provision of TTNS equipment and disposables in self-managed OAB would ensure health equity in this healthcare setting. Related to cost were negative attitudes when TTNS impacted on work due to strict healthcare system appointment times; this is something which self-managed TTNS circumvents.

Approaches to improving adherence and long-term motivation for OAB treatment should consider the efforts required by the individual and degree of behavioural change needed [13]. Our study shows the behavioural change required for home-treatment to be low. Studies of TTNS ‘therapy’ to date have shown significant improvements using a variety of session delivery frequencies, ranging from once weekly to daily [4]. The strong safety profile of this treatment provides reassurance that self-management is a safe, viable approach to OAB treatment by women [4].

A limitation of this study is that participants were recruited from women referred to secondary care specialist clinics, although all were still undergoing conservative management. Results are therefore likely transferable to different healthcare settings but we accept that findings from a primary care cohort may be different.

## Conclusions

This paper addresses a gap in our understanding of women’s experiences of TTNS as a treatment for OAB and their views on self-management using this approach. It highlights acceptability of home TTNS long-term, in terms of ease of use, flexibility and convenience. The positive experiences of women using TTNS at home adds to the accruing evidence for the inclusion of TTNS as a non-invasive, self-management strategy in the OAB treatment pathway.

## Data Availability

The datasets used and/or analysed during the current study available from the corresponding author on reasonable request and with permission of the Sponsor (NHS Greater Glasgow & Clyde Clinical Research and Development department).
